# Sociodemographics, functional ability, chronic conditions, and loneliness in older US adults in the 2020 wave of HRS

**DOI:** 10.1093/geroni/igaf122.2715

**Published:** 2025-12-31

**Authors:** Laurie Theeke, Lashawn Hutto, LaTonya Smith

**Affiliations:** The George Washington University, Washington, District of Columbia, United States; The George Washington University, Washington, District of Columbia, United States; The George Washington University, Westfield, New Jersey, United States

## Abstract

Loneliness is a known determinant of poor health outcomes and a priority for community-dwelling older adults. This analysis describes relationships among loneliness, determinants of health, functional ability, and chronic illnesses for community-dwelling adults 50 and older in the US during 2020. SPSS was used for analysis on the Rand longitudinal data file with 4,520 cases (60.1% female) selected from Wave 15 who complete data on the UCLA-11 The mean age was 69.23 (SD 10.06), with 71.1% reported as Caucasian and 19.2% reported as Black. Majority (86.1%) were reported as not Hispanic and married (57.1%) and 82.6% had completed high school or higher. The average annual household income was less than $20,000 and 34.2% reported ongoing financial strain with 19.5% reported housing problems. Participants averaged 8.66 (SD 12.74) doctor visits since 2018 and over 2 days per month with family helpers. The median was 2 chronic conditions (range 0-8), with high prevalence for arthritis (63.3%), hypertension (62.7%), diabetes (26.9%), heart disease (24.4%), and cancer (17%). Functional impairment for basic ADLs was low except for 33.3% reporting difficulty getting up from a chair but 27.7% reported trouble walking several blocks and 14.2% reported difficulty walking one block. Though 52.2 % reported smoking in their lifetime, only 9.4% reported current smoking. Significant correlations of loneliness (p < .001) included depression (CES-D scores), fine and gross motor impairment, and BMI. Wellness and prevention interventions that are community-based would have potential to diminish chronic conditions and emphasize functional ability which could diminish likelihood of loneliness.

